# Detecting Aflatoxin B_1_ in Peanuts by Fourier Transform Near-Infrared Transmission and Diffuse Reflection Spectroscopy

**DOI:** 10.3390/molecules27196294

**Published:** 2022-09-23

**Authors:** Wanqing Yao, Ruanshan Liu, Fengru Zhang, Shuang Li, Xiaoxia Huang, Hongwei Guo, Mengxia Peng, Guohua Zhong

**Affiliations:** 1 Key Laboratory of Conservation and Precision Utilization of Characteristic Agricultural Resources in Mountainous Areas, School of Chemistry and Environment, Jiaying University, Meizhou 514015, China; 2 School of Science, Harbin Institute of Technology, Shenzhen 518055, China; 3 Key Laboratory of Integrated Pest Management on Crops in South China, Ministry of Agriculture and Rural Affairs, South China Agricultural University, Guangzhou 510642, China

**Keywords:** FT-NIR, peanut, principal component analysis (PCA), spectral acquisition module, aflatoxin B_1_

## Abstract

Aflatioxin B_1_ (AFB_1_) has been recognized by the International Agency of Research on Cancer as a group 1 carcinogen in animals and humans. A fast, batch, and real-time control and no chemical pollution method was developed for the discrimination and quantification prediction of AFB_1_-infected peanuts by applying Fourier transform near-infrared (FT-NIR) coupled with chemometrics. Initially, the near-infrared transmission (NIRT) and diffuse reflection (NIRR) modules were applied to collect spectra of the samples. The principal component analysis (PCA) method was employed to extract the characteristic wavelength, followed by different preprocessing methods (seven methods) to build an effective linear discriminant analysis (LDA) classification and partial least squares (PLS) quantification models. The results showed that, for both the NIRT or NIRR modules, the LDA classification models satisfactorily distinguished peanuts infected with AFB_1_ or from those not infected, with external validation showing a 100% correct identification rate and a 0% misjudgment rate. In addition, combined with the concentration of AFB_1_ in peanuts determined by enzyme-linked immunoassay assay, the best partial least squares (PLS) models were established, with a combination of the first derivative and the Norris derivative filter smoothing pretreatment (*R*_c_^2^ = 0.937 and 0.984, RMSECV = 3.92% and 2.22%, RPD = 3.98 and 7.91 for NIRR and NIRT, respectively). The correlation coefficient between the predicted value and the reference value in the external verification was 0.998 and 0.917, respectively. This study highlights that both spectral acquisition modules meet the requirements of online, rapid, and accurate identification of peanut AFB_1_ infection in the early stages.

## 1. Introduction

Peanuts, as one of the most important oil crops, are cultivated at a large scale all over the world. The yield of peanuts ranks the first among the oil crops in China, accounting for 40% of the world’s total peanut production [1]. Peanuts are rich in oil, proteins, fibers, unsaturated fatty acids, vitamins, and minerals [2], thus being a natural medium for fungal growth. Once the environment is suitable, the fungus will produce aflatioxins in the field or under storage conditions [3]. Aflatioxin is the most concerned and commonly known mycotoxin in the world, threatening human and animal health. It is also the most important risk factor to be solved for the safe consumption and export of agricultural products such as grain and oil crops [4]. Aflatioxin B_1_ (AFB_1_) is the most toxic and widely distributed mold of aflatoxin [5]. Due to aflatoxin’s deleteriousness and frequent occurrence in agricultural products, research on aflatioxin reduction and elimination has gained global attention. Generally, in China [6] and the USA [7], the accepted aflatoxin level in food is 20 ppb.

Nowadays, different conventional methods are used to obtain reference data, such as thin-layer chromatography (TLC), high-performance liquid chromatography (HPLC), liquid chromatograph mass spectrometry (LC-MS), and enzyme-linked immunosorbent assay (ELISA). All these methods are generally difficult, expensive, time-consuming, and unsuitable for real-time control measures, despite having high accuracy, sensitivity, and dependability [8]. Conversely, spectroscopy technology has the advantages of no sample pretreatment, short determination time, no use of chemical reagents, and simultaneous determination of multiple components [9,10], providing a new way for the rapid screening, qualitative discrimination, or highly sensitive detection of mycotoxins in grains. It is complementary to the detection of large and precise physical and chemical analysis instruments [11]. Its use with chemometric analyses has shown great potential and advantages in the detection of food and feed mycotoxins, such as common toxigenic fungal species in corn [12], maize [13], paddy rice [14], brown rice [15], peanuts [16,17,18], and rice [19].

In the last decade, most of the NIR models developed by previous scholars have been based on surface reflection patterns of solid forms of grains or seeds. However, aflatoxin typically infects the kernel germ, which likely affects the chemical and optical properties [3,8], and can leave little indication of its presence on the kernel surface [20]. Pearson et al. [21] indicated that the distribution of mycotoxin in a grain pile is uneven, and the contamination is random. Moreover, Li et al. [22] reported that there are differences among non-uniform solid particles, with spectra containing both the type of information to be extracted for analysis and the individual information to be eliminated. FT-NIR spectroscopy is a technology that, by use of an interferometer, further improves the spectral reproducibility and the accuracy and precision of wavelength discrimination [23]. Lee et al. [13] reported that FT-NIR spectroscopy can be an alternative method for aflatoxin detection in maize. Huang Xingyi et al. [24] established the identification model of moldy and budding peanuts by FT-NIR combined with the KNN identification method. FT-NIR spectroscopy has advantages over dispersive NIR spectroscopy with higher instrument stability, light penetration depth, and predictive power for some quality characteristics [25]. However, there are very few studies on contaminated peanuts based on FT-NIR spectroscopy. Conversely, based on previous reports describing the strengths and criticality of the application of NIR spectroscopy in the quantification or discrimination of mycotoxins, an approach is proposed herein to develop discrimination and quantitative models, with a special focus on low AFB_1_ contamination level in peanuts, to establish discrimination and quantitative models using two FT-NIR spectral acquisition modules (NIRT and NIRR), in combination with different preprocessing and machine learning methods. This article may provide technical support and a reference basis for rapidly evaluating the AFB_1_ contamination risk of agricultural products.

## 2. Results

### 2.1. Analysis of a Reference Aflatoxin B_1_ (AFB_1_) Concentration

**Figure 1:**
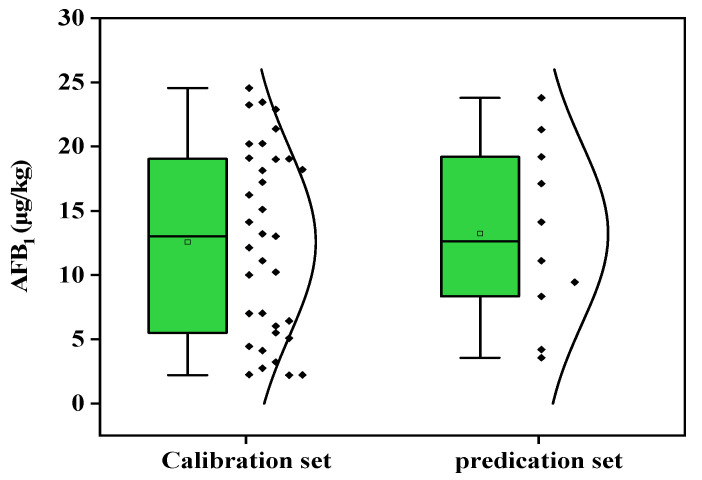
Distribution and reference AFB_1_ concentrations in peanuts for calibration and validation sets.

Since there were low concentrations of early infection in the samples in production, the AFB_1_ contamination levels of the peanut samples are shown in Figure 1, fluctuating within the permissible concentration of aflatioxin in grains issued by China and the USA (20 μg/kg) as the reference, for the purpose of early detection in production. The reference covers the concentrations of total aflatoxin, ranging from 2.21 to 23.79 μg/kg, with an average of 12.59 μg/kg and a variation range of 56.71%. The NIR calibration model was based on these samples, and the external validation set was within the range of the calibration set, which can effectively achieve predictions.

### 2.2. FT-NIR Spectra of Positive Samples

**Figure 2:**
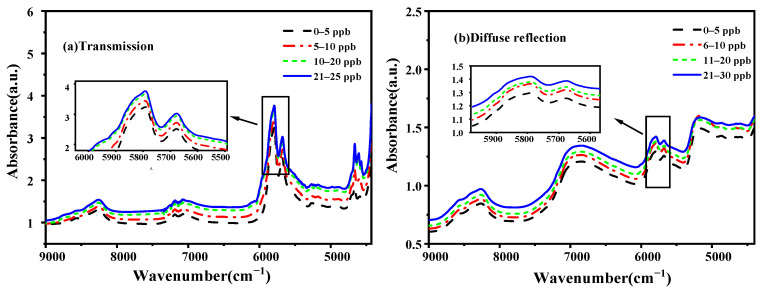
Average FT-NIR spectra of the positive samples.

In Figure 2, spectra from the NIRT (Figure 2a) and NIRR (Figure 2b) modules with different AFB_1_ concentrations in the range of 9000–4000 cm^−1^ are shown. The spectra from NIRT (Figure 2a) showed sharp, strong absorption peaks at wavenumbers 5681 and 5819 cm^−1^. The spectra from NIRR (Figure 2b) had a similar shape, showing several different peaks and a generally growing trend, related to absorbance values, that increased with an increasing wavenumber.

**Figure 3:**
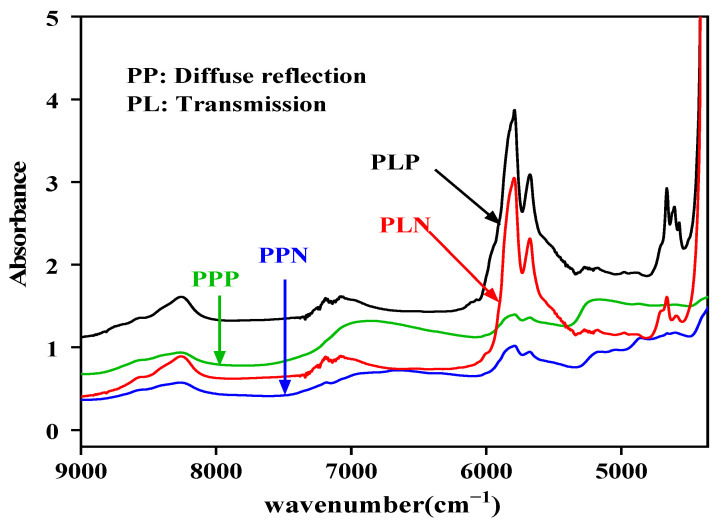
Average FT-NIR spectra collected by the diffuse reflection and transmission modules.

For both the NIRT and NIRR modules, the positive samples of the NIR spectra of the integral absorbance value were stronger (Figure 3). Regardless of whether the samples were contaminated or not, the spectra collected by the transmission modules (PLP and PLN) showed sharp, strong absorption peaks between 5500 and 6000 cm^−1^.

### 2.3. PCA Analysis of Principal Components

**Figure 4:**
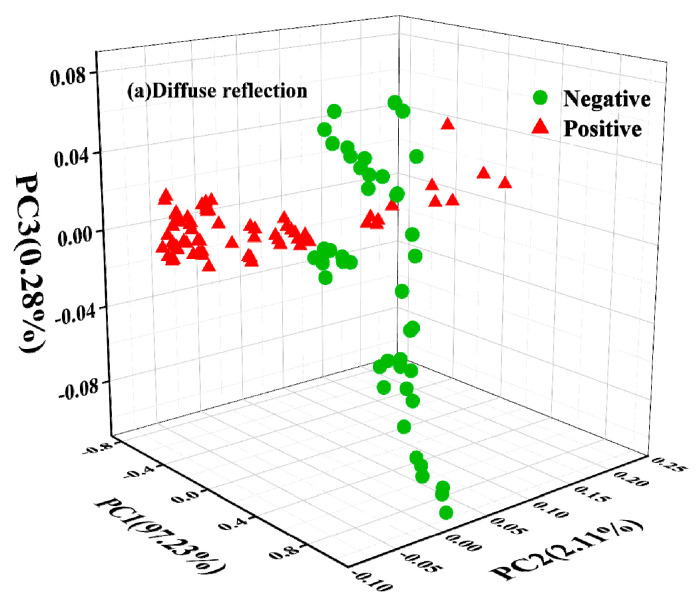

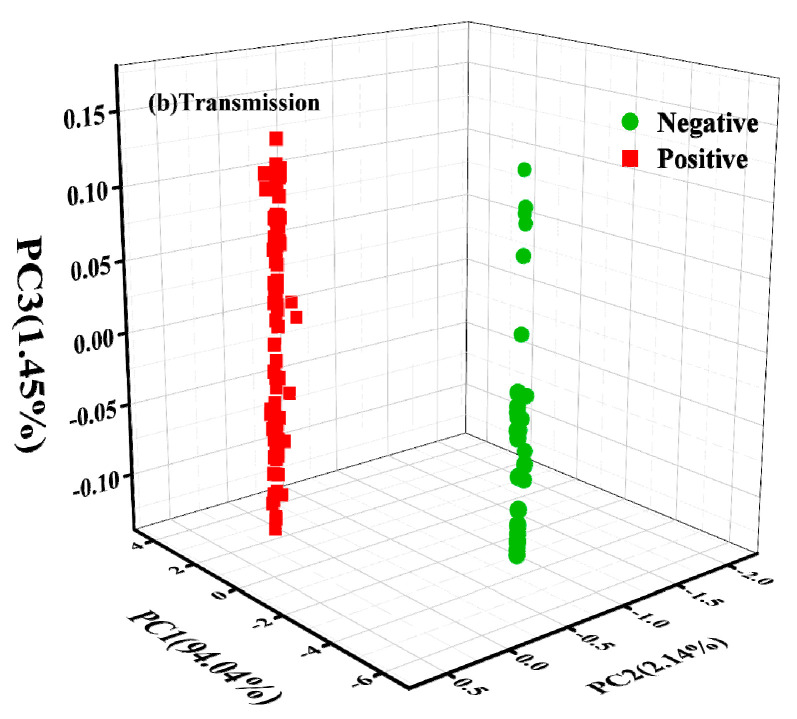
Scores of the first three principal components.

The accumulative contribution rates of the first three PCs are shown in Figure 4, with 99.62% and 97.63% from the spectra collected by the NIRR and NIRT modules, respectively. The three-dimensional spatial clustering in Figure 4 also proves the feasibility of PCA for distinguishing between contaminated and non-contaminated samples. In addition, it can be seen from the comparison that the clustering effect of the transmission module of oil samples was better than that of the diffuse reflection module of the powder samples, with no crossover between different samples, but the reflection module partially overlapped. Overall, it means that the first three PCs could completely represent all the information of the original spectra.

**Figure 5:**
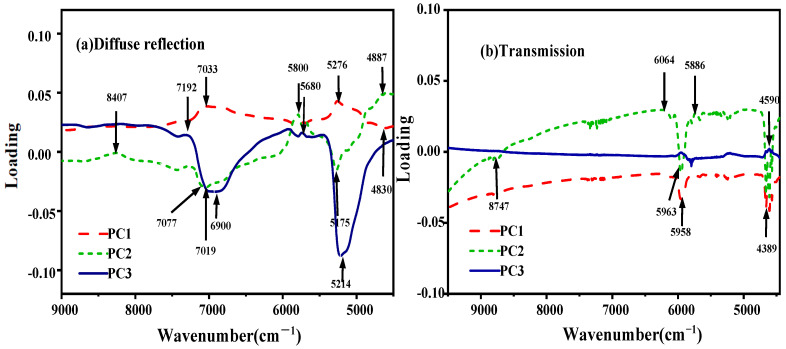
PCA score loading diagram of the sample.

PCA loading can be used to extract the characteristic wavelength, with either the local highest or lowest peak of the loading curve being considered as a characteristic band [26]. Figure 5 shows the PCA loading curves of the first three principal components (PC1, PC2, and PC3) in detail, among which the peaks and valleys deviating from the horizontal line were effective in determining the degree of peanut mildew [27]. As can be seen in Figure 5, for both the NIRR and NIRT modules, there were valleys and peaks in the region of 4887–4389 cm^−1^, related to the stretching vibration of the N–H group in the amino acid [28], while the peaks in the region of 6064–5680 cm^−1^ could be assigned to the second-order frequency-doubling stretching vibration of the -CH_2_- group in the fatty acid [3,14]. The peaks at 8407 and 8747 cm^−1^ were the second-order frequency doubling of the C-H stretching vibration of aliphatic hydrocarbons [29,30]. The C-H bonds of the aromatic ring influenced the diffuse reflection module in the range of 7274–6900 cm^−1^ [15,30]. Some of the spectral values in the literature are close to our results.

Table 1 and Table 2 summarize the bands and vibration modes of the chemical bonds in the samples, respectively, which were selected as the modeling wavelengths of the diffuse reflection and transmission module.

### 2.4. Determination and Validation of LDA Discrimination Models

#### 2.4.1. Determination of LDA Models

**Figure 6:**
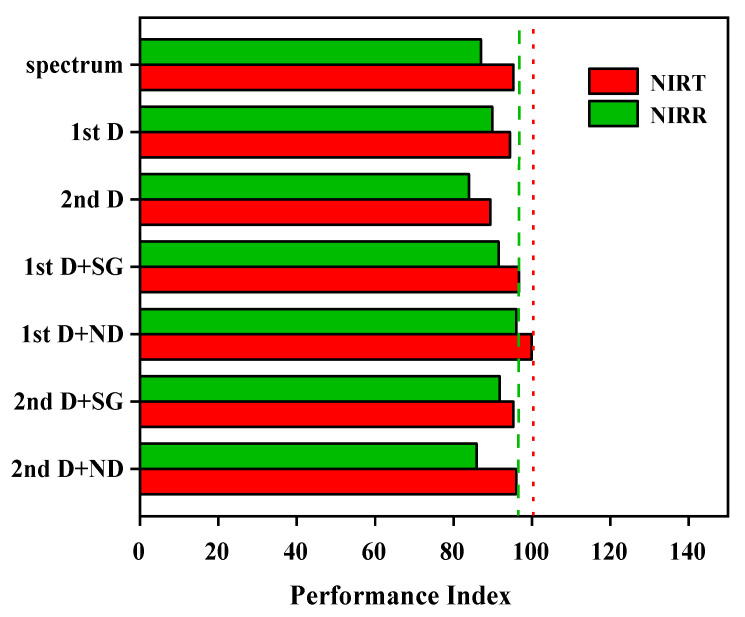
Performance indices of the different data-processing methods.

**Figure 7:**
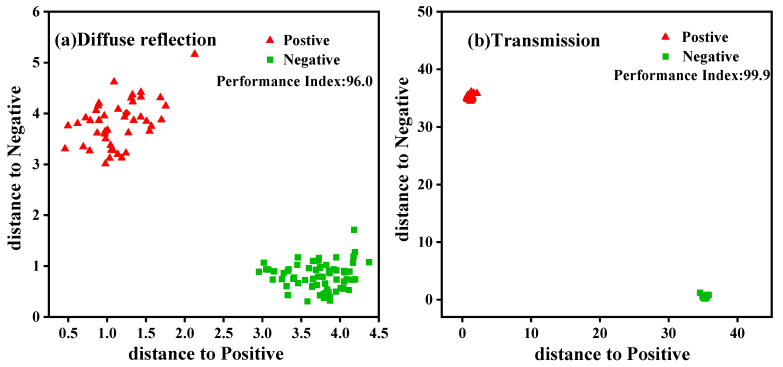
Principal component Mahalanobis distance discriminant analysis models.

Based on the single and combined pretreatment methods presented in Section 2.3 the first three PCs’ scores extracted from the PCA analysis were used to establish an LDA discrimination model combined with the Mahalanobis distance. The results are shown in Figure 6, where the models pretreated by “1st D + Nd” had high differentiation and robustness, with the highest performance indexes of 96 (NIRR module) and 99.9 (NIRT module). Further LDA classification models were established by 1st D + Nd pretreatment, as shown in Figure 7, and clustering of non-contaminated groups, as well as contaminated groups, can be clearly observed in each module.

#### 2.4.2. External Evaluation of LDA Models

**Figure 8:**
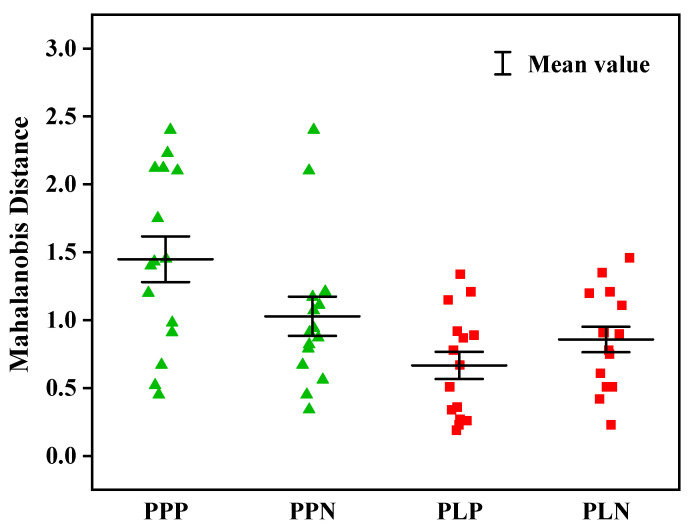
External verification results of the LDA identification models.

The external validation set, including 15 positive and 15 negative samples, was imported into the LDA models for verification. As can be seen in Figure 8, the Mahalanobis distance of the validation set was less than 3, with a correct identification rate of 100%. No false negatives or positives were observed, and the misjudgment rate was 0%. The model quickly and accurately identified the occurrence of AFB_1_ infection in peanuts. A slightly smaller Mahalanobis distance and a better clustering effect of the NIRR module of the oil samples was obtained compared to the NIRT module of the powder samples. Overall, it could be inferred from the results that the LDA models established by the NIRR and NIRT modules can be used for qualitative analysis of AFB_1_ contamination in peanuts, with the NIRT module being slightly superior to the NIRR module to a certain extent.

### 2.5. Determination and Validation of PLS Quantification Models

#### 2.5.1. Determination of PLS Models

**Figure 9:**
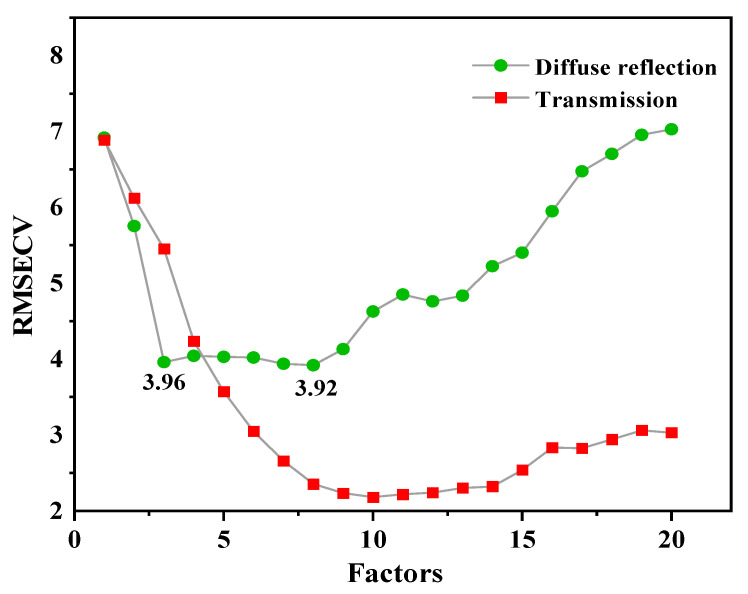
Variation of the factor numbers using different detection methods.

The ideal number of latent variables (LVs) was determined at the minimum of the predicted root mean square error of cross-validation (RESECV) [28]. Generally, good calibration statistics were obtained with the ideal number of LVs for peanut samples contaminated with AFB_1_. As can be seen from Figure 9, the number of LVs of the diffuse reflection and transmission modules allowed to enter the PLS models was set to 8 and 10, respectively.

PLS models (Table 3) with different preprocessing methods were built using characteristic wavelengths and the ideal number of LVs to predict the AFB_1_ contamination level in peanuts, and the models were evaluated according to the *R*_c_^2^, RMSE, and RPD. Comparing the performance of these models, the best accuracy and prediction was achieved with a combination of the first derivative with Nd smoothing (1st D + Nd) preprocessing, which demonstrated good predictive ability, with *R*_c_^2^ = 0.937, RMSEC = 2.51%, and RPD = 3.98 for the NIRR model and *R*_c_^2^ = 0.984, RMSEC = 1.28%, and RPD = 7.91 for the NIRT model.

**Figure 10:**
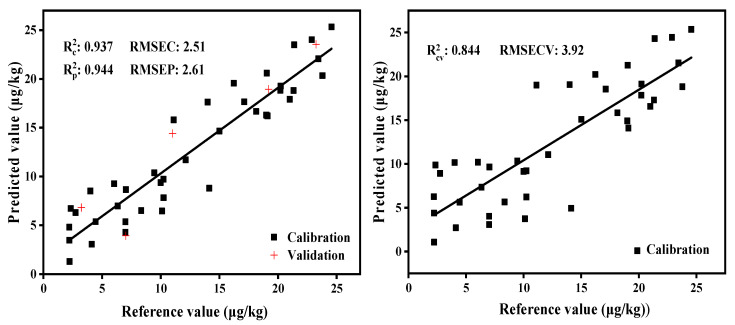
PLS model and cross-validation of the diffuse reflection module.

**Figure 11:**
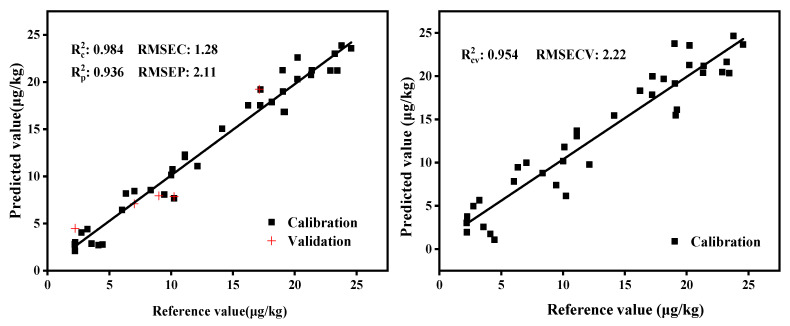
PLS model and cross-validation of the transmission module.

PLS was used for modeling after optimizing the number of factors and preprocessing methods. It can be observed from Figure 10 and Figure 11 that the PLS models of the transmission module yielded higher predictive precision and better regression quality, with the determination coefficient and RMSECV for the calibration and validation sets being 0.984 and 2.22%, respectively, while they were 0.937 and 3.92% for the diffuse reflection module. The two models can be used for process or quality control, because the value of RPD is greater than 3 [31].

#### 2.5.2. External Evaluation of the PLS Models

**Figure 12:**
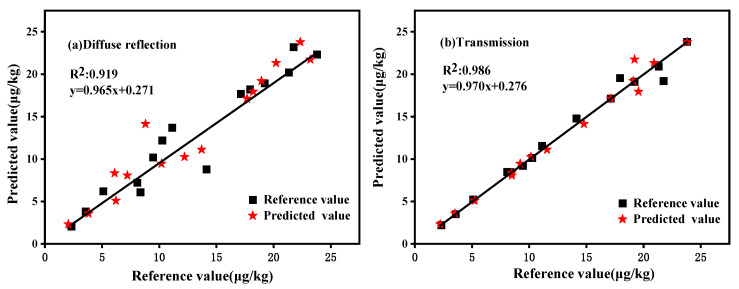
External validation of the reference and predicted concentrations of AFB_1_ in peanuts.

To evaluate the robustness of the PLS models, 30 unknown samples were used for external validation. The spectra obtained from the diffuse reflection and transmission detection were entered as input into the calibration equation and prediction was performed. The prediction set and reference values can be seen in Table 4, with the absolute values of the relative deviations for the diffuse reflection and transmission modules being between 1.35% and 37.72% and 0.06% and 11.60%, respectively. The relatively high relative deviation values are related to the small values of the AFB_1_ concentration. Figure 12 shows the prediction results of the PLS models for all of the external validation set samples. The samples were distributed on both sides of the center line, showing a high degree of correlation. The determination coefficients (*R*^2^) of the predicted and reference values of the PLS models of diffuse reflection and transmission were 0.919 and 0.986, respectively.

The values obtained from the prediction were also compared using Student’s *t*-test, which provides a statistical test of whether or not the means of two groups are equal. Both levels of significance (*p*-values) resulted less than t_0.05_, *p* > 0.05, indicating a satisfactory predictive ability and confirming the potential of FT-NIR analysis for AFB_1_ prediction in peanuts.

## 3. Discussion

The NIR spectra of the integral absorbance value of contaminated peanuts is stronger, probably because the infection causes mildew of the starch of peanuts, as well as sugar, lipid, and protein changes, in addition to mold, which starts producing metabolites, causing different degrees of change in the spectra [7,29]. Thus, it indirectly indicates the content of toxins in peanuts [31,32]. The spectra collected by the diffuse and transmission detection modules were different. The spectra showed sharp, strong absorption peaks in the transmission module, which might be because transmitted light can penetrate through the sample to be measured and can reach deep inside the sample, and it contains deeper information about said sample. While in the diffuse reflection module, the NIR light source cannot completely penetrate solid particles, and the spectrum only carries the information of one side or epidermis of the solid sample due to the influence of the placement position. This behavior was also observed by Li Hao Guang [22]. These spectral peaks may relate to the chemical and physical properties of the samples.

Effective feature wavelengths extraction for spectral differences is very crucial for classification and quantification of aflatoxin levels in peanut samples using chemometric methods. PCA was applied for spectral data dimensionality reduction to identify the characteristic wavelength. According to the PCA scoring loading analysis, the spectral information was affected by spectrum acquisition methods. Diffuse reflected light is the light processed by the light source that enters the interior of the sample and returns to the surface of the sample after multiple reflections, refractions, diffractions, and absorptions [23]. Consequently, there were more peaks and valleys. Compared to previous research by Gaspardo et al. [23], Daniel Kimuli et al. [7], and Fei Shen et al. [28], except for those common wavelengths, there were some differences for the characteristic wavelengths selected as the modeling wavelengths in our research. Near-infrared spectrum technology relies on the statistical analysis of a given set of data, and it is normal for different spectral acquisition methods to have some differences in the selected wavelengths [3]. Thus, the existence of spectrum differences may be caused by the different spectrum acquisition methods, which provided the basis for establishing classification and quantification models of FT-NIR spectroscopy.

Spectral pretreatments were applied to eliminate the influence of high-frequency random noise, baseline shift, and sample heterogeneity. The LDA classification models, involving pretreatment with the first derivative combined with Norris derivative filter smoothing (1st D + Nd), showed the best differentiation and robustness. It is possible that it reduces additional effects such as the baseline offset and slope of the spectrum, and improves the resolution and sensitivity of the spectral data [33]. The clustering of different groups suggests that samples of the same cluster may have similar physical or chemical characteristics [7]. In both spectrum acquisition methods, high efficiency values were obtained in the discrimination and classification of the samples in Figure 7. It is noteworthy that the classification accuracy of the transmission model was better than that of the diffuse reflection model, for which the feature space variance of the aflatioxin- positive and -negative groups was lower and better separated. Pearson et al. [20] also obtained better results in the detection of aflatioxin in corn kernels by visible region transmission spectroscopy, with a classification accuracy of 95%. This may be because overcoming stray light is difficult for the diffuse reflection module [34]. Liu Yande et al. [35] reported that the diffuse reflection module only carries the information of one side or epidermis of a solid sample, which may be the reason for the slightly lower accuracy. Based on the transmission module of NIR, a discriminant model of peanuts infected by a variety of mushy mold was established by Liu Peng et al. [30], and the discriminant accuracy was 99.17%, which may be affected by external factors such as non-uniform placement of grain samples. However, the prediction accuracy of our models was improved, probably because the instrument’s integrating sphere already reduced scattering and enhanced the effect of molecular absorption [23]. The classification accuracy was 100% for the external validation set for the two LDA models. The accuracy of the classification needs to be verified in a broader sample database in future studies to make the model more stable.

The best PLS models (with *R*_c_^2^ = 0.937 and 0.984 for NIRR and NIRT, respectively) were established for predicting the AFB_1_ content in peanuts, with the appropriate number of latent variables (LVs) and the pretreatment method. The appropriate number of LVs of the diffuse reflection and transmission modules allowed to enter the PLS models was set to 8 and 10, respectively, presenting as much useful information as possible, with less noise and over-fitting avoidance [36]. The models were stable and achieved satisfactory accuracy. The combination of 1st D + Nd smoothing pretreatment was found to be more effective compared to any other chemometric method, for both the NIRR and NIRT spectrum acquisition methods. This may be because the derivative eliminates baseline drift and improves the spectral resolution. However, we found that the models pretreated with second derivative processing were poor, as they might increase the noise level and reduce the spectral signal-to-noise ratio in some case [28]. The PLS model of the transmission module yielded higher predictive precision and better regression quality. Similar results were also obtained by Pearson et al. [20] when studying aflatioxin contamination in single-corn kernels by employing transmittance and reflectance spectroscopy. They stated that light passes through the kernel, ensuring that the constituents inside the kernel have an opportunity to interact with the NIR radiation during transmittance spectroscopy. Meanwhile, in the diffuse reflectance module, some energy cannot penetrate the kernel and is reflected back to the sensor.

External evaluation of the PLS models presented that the prediction accuracy of the diffuse reflection model was slightly poor, consistent with the conclusion that the ability of the diffuse reflection spectrum to reflect sample information was worse than that of transmission. The model needs to be further optimized and can only be used for rough detection. The results obtained were similar to those of Berardo et al. [37]. They developed a PLS model of maize with *R*^2^ = 0.80 and concluded that it could only be used for the rough screening of Fusarium verticillioides-infected maize. Shen et al. [19] obtained slightly higher determination coefficient values (0.8823) for rice contaminated with harmful mold infection. In our study, the predicted value of the transmission model had a good correlation with the chemical reference value, and the accuracy was good and robust, which was suitable for the early identification of moldy peanuts. Subsequent explorations will take into account sample preparation, scan area, feature extraction, and analysis efficiency to improve the predictive power of models built from diffuse reflectance spectroscopy.

## 4. Materials and Methods

### 4.1. Aspergillus Flavus Spore Suspension Preparation

According to the method described by Jing Dan et al. [38], strains were isolated from moldy peanuts and identified as Aspergillus flavus by the Microbial Analysis and Testing Center in Guangdong Province, China. Cultures of the strains were incubated at 28 °C and 85% relative humidity (RH) for six days on potato dextrose agar (PDA) medium to produce large numbers of spores. After incubation, the spores were harvested and slowly rubbed with a sterile stainless steel inoculation loop. Subsequently, the suspension obtained was filtered through sterile gauze for further use. The concentration of spores was determined using the standard pour plate method [15], suspended in sterile distilled water at a dilution of 1.6 × 10^6^ CFU/mL.

### 4.2. Peanut Sample Inoculation

The peanuts (No. 1 of Zhongkaihua) were refrigerated for later use after irradiation sterilization. The sterilized peanuts were soaked in the solution of spores and stirred for 5 s. Next, they were placed an incubator at 30 °C and 85% RH for 11 days for aflatioxin production. On the third day after artificial inoculation of AFB_1_, 12 samples (80 g of peanut per sample) were collected every other day for analysis. A total of 60 samples were collected five times, representing the positive samples, while the sterilized peanut samples represent the negative samples.

### 4.3. ELISA Analysis of Aflatoxins

The reference AFB_1_ concentrations of the positive samples were detected by Enzyme Linked Immunosorbent Assay (Multiskan SkyHigh, ELISA kit, Thermo, Waltham, MA, USA) according to the national standard of China (GB/T 5009.22-2016) [39]. ELISA is a semi-quantitative assay capable of detecting AFB_1_ levels from 1 to 50 ppb. These reference AFB_1_ concentrations were used for model establishment and validation.

### 4.4. Sample Preparation for NIR Analysis

All samples were crushed into homogenized powder using a No. 60 mesh sieve and stored at 4 °C. In this case, 60 positive samples of peanut powder (PPP) were obtained. After collecting the NIRR spectroscopy, the samples were pressed into liquid oil by a hot press and clarified peanut oil from the upper layer was obtained as positive peanut oil samples (i.e., positive sample of peanut liquid, PLP). Here, 60 sterilized peanut powder (i.e., negative sample of peanut powder, PPN) and 60 sterilized peanut oil (i.e., negative sample of peanut liquid, PLN) samples represent the negative samples. According to the Kennard-Stone (K-S) algorithm, the samples were divided into a calibration set and a prediction set, as shown in Table 5. To remove the effect of moisture on the spectrum, all contaminated and negative samples were dried at 40 °C for 6 h to ensure that the moisture content in the samples was below 15% [15].

### 4.5. Spectra Acquisition

Fourier transform spectra of the 240 samples were acquired using an Antaris II FT-NIR spectrometer (Thermo Nicolet, Waltham, MA, USA) equipped with an interferometer, a long-life light source, and an InGaAs detector. In the NIRR module, around 12 g of the peanut powder sample was uniformly packed into a integration sphere with an adapter spinner for sample rotation and directly placed on the sample holder for measurement. At the same ambient temperature and humidity, in the NIRT module, around 1 mL of peanut oil was loaded into a square quartz liquid tank with an optical path of 2 mm. In the region of 10,000–4000 cm^−1^, measurements were conducted with a resolution of 8 cm^−1^ for 64 scans to ensure an adequate signal-to-noise ratio. The air background was taken each hour. All of the samples were run in parallel five times and were scanned in triplicate for each parallel sample. A total of 15 NIR spectra were obtained for each sample. The average spectrum of each sample was considered as the final spectral data for participating in modeling.

### 4.6. Multivariate Calibration

All the statistical analyses were conducted using TQ Analyst and Omnic (Version 9.0, Thermo Electron Corp., Waltham, MA, USA) in this study. The purposes of statistical analyses were: (1) To select a characteristic wavelength range, (2) to extract feature information from the NIRR and NIRT spectra, (3) to establish a classification model to classify AFB_1_-contaminated peanuts, and (4) to predict the AFB_1_ concentration of contaminated peanuts.

Principal component analysis (PCA), linear discriminant analysis (LDA), and partial least squares (PLS) regression have been proven to be effective in many applications [15] [40].

At present, numerous feature extraction methods have been proposed and applied to dimensionality reduction of spectral data in literature reports, such as Genetic algorithm (GA), Successive projections algorithm (SPA), Principal component analysis (PCA), Competitive adaptive reweighted sampling (CARS), etc. These methods have a solid theoretical foundation and are easy to implement and analyze, which have been used successfully in many applications. PCA is commonly applied, as a tool for data dimensionality reduction, to spectral data to investigate the presence of spectral variation among samples [3,7,13,28]. The spectra were mean-centered, and selection of variables was carried out for identification of the most relevant wavelengths, thereby reducing correlated variables [41]. The data were summarized by altering the original variables into a new set of linearly irrelated variables called principal components (PCs). The cumulative contribution rate represents the ability of the corresponding PC to interpret the original variable. Additional information about the characteristic wavelengths can also be provided by the load at each wavelength in the corresponding PC [42].

LDA, widely recognized as valuable for classification problems [43], is a supervised pattern recognition method based on the class model combining PCA and the Mahalanobis distance (MD). Through multivariate analysis technology, a full spectrum is not provided as a predictor because the number of predictors must be less than the number of responses. Therefore, the loading obtained by stepwise regression and PCA is used to select the most relevant wavelengths and eliminate the relevant variables to compare the obtained results [41].

PLS is a classical linear modeling method that compresses spectral data into an orthogonal structure of a small number of orthogonal factors called latent variables (LVs) [41]. LVs describe the maximum covariance between spectral information and the reference content value. The established quantitative prediction model has the advantages of comprehensive screening of spectral data, full extraction of effective spectral information of samples, consideration of internal connections, etc.

### 4.7. Spectral Preprocessing

Many data-processing methods can be applied to eliminate the influence of high-frequency random noise, baseline shift, and sample heterogeneity. These methods include the derivative smoothing filter (Norris derivative filter, Nd), the convolution smoothing filter (Savitzky-Golay, SG), the first derivative (1st D), and the second derivative (2nd D). More useful information could be obtained after pretreatment. In our current research, a total of seven methods independently or in combination were selected for composite spectral preprocessing, and the best spectral preprocessing method was finally selected.

### 4.8. Model Establishment and Evaluation

The classification model was implemented by linear discriminant analysis combined with principal component analysis (PCA–LDA), comparing the effect of different preprocessing methods based on the performance index and classification accuracy of the calibration and validation sets. In the PCA–LDA method, PCA was first used to reduce the dimensionality of the spectral data and determine the characteristic wavelength; next, LDA was performed on the first few PCs with a larger contribution rate to establish the classification model. PLS models were used to predict the AFB_1_ concentrations in the peanut samples. The ideal model was evaluated by the determination coefficient (*R*_c_^2^), root mean square error (RMSE), and relative percent deviation (RPD) [44]. The performance of the estimation models was further analyzed using an independent test set called the validation set, with samples not included in the original models.

### 4.9. Data Processing

The data measured by ELISA were imported into SPSS 25.0 data-processing software, and the mean value, amplitude, and coefficient of variation of the data were calculated. An independent samples *t*-test was used to analyze the prediction deviation of the NIR prediction model under the two detection methods. The analysis results are expressed as *p*-values, with *p* < 0.05 indicating a significant difference and *p* < 0.01 highlighting an extremely significant difference [39].

## 5. Conclusions

Fourier transform near-infrared transmission and diffuse reflection spectroscopy were employed to detect AFB_1_ contamination in naturally infected peanuts. The influence of the characteristic wavelength and various pretreatment methods on the detection accuracy was investigated, respectively. The results showed that the LDA models established by the two modules could quickly and accurately identify AFB_1_ infection in peanuts. The PLS quantitative model established by the NIRT module was litter superior to that of the NIRR module. The proposed methodologies may provide a reference for the detection of peanut mycotoxin contamination by FT-NIR in industrial production, which have practical applications for screening peanut samples and for preventing moldy peanuts from entering the food chain. If the number of samples could be expanded and a more reasonable chemometric algorithm could be involved in subsequent studies, the models will have greater utility and robustness.

## Figures and Tables

**Table 1:** Functional bonds and vibration modes in the NIRR module.

Wavenumber (cm^−1^)	Functional Bonds	Vibration Modes
7274 and 7033 cm^−1^	Arene methyl C–H	Telescopic vibration combination frequency [29]
6900 cm^−1^	Aromatic amine N–H	Scale one frequency doubling [3,15,19,28,30]
5800 cm^−1^	Fat hydrocarbon C–H	Stretching vibration [15,19]
5680 cm^−1^	Aliphatic hydrocarbon –CH_2_–	Symmetry vibration [3,14]
5175 and 5214 cm^−1^	Water molecules O–H and HOH	Combination frequency of stretching and bending vibration [3,19,28,30]

**Table 2:** Functional bonds and vibration modes in the NIRT module.

Wavenumber (cm^−1^)	Functional Bonds	Vibration Modes
8747 cm^−1^	Aliphatic hydrocarbon C–H	Stretching vibration double frequency [29,30]
5958 cm^−1^	Aromatic C–H	Telescopic first-order vibration [14]
5819 cm^−1^	Aliphatic hydrocarbon C–H	First-order frequency doubling of stretching vibration [30]
5742 cm^−1^	Arenes attached to methylene C–H	Telescopic first-order vibration [14]
4590 cm^−1^	Protein N–H	Telescopic vibration combination frequency doubling [28,30]
4389 cm^−1^	Starch C–H, CH_2_ Alcohol group O–H	Expansion and deformation [3,15,19]

**Table 3:** Descriptive statistics of the PLS models for the calibration and validation sets.

Spectral Detection Module	Pretreatment Methods	LVs	Calibration		Validation		RMSECV (%)	RPD
			*R*_c_^2^	RMSEC (%)	*R*_p_^2^	RMSEP (%)		
Diffuse reflection	Spectrum	2	0.884	3.34	0.967	2.28	3.70	2.94
	1st D	3	0.888	3.29	0.788	4.85	4.42	2.99
	2nd D	1	0.566	5.90	0.962	5.00	8.50	1.52
	1st D + SG	3	0.890	3.26	0.882	4.00	4.28	3.52
	1st D + Nd	8	0.937	2.51	0.944	2.61	3.92	3.98
	2nd D + SG	1	0.357	6.69	0.540	6.42	7.11	1.25
	2nd D + Nd	4	0.877	3.64	0.926	3.62	4.18	2.85
Transmission	Spectrum	10	0.971	1.74	0.797	4.50	2.78	5.87
	1st D	7	0.857	3.76	0.377	6.76	5.14	2.83
	2nd D	1	0.663	5.47	0.914	3.08	6.24	1.72
	1st D + SG	4	0.736	4.95	0.942	2.35	5.87	1.95
	1st D + Nd	10	0.984	1.28	0.936	2.11	2.22	7.91
	2nd D + SG	1	0.610	5.79	0.855	3.17	6.23	1.60
	2nd D + Nd	7	0.864	3.69	0.937	3.39	4.88	2.71

**Table 4:** Prediction results of the PLS models.

Spectral Detection Module	Reference Value (μg/kg)	Predicted Value (μg/kg)	Absolute Deviation (μg/kg)	Relative Deviation (%)
Diffuse reflection	11.11	13.7	−2.59	−23.31%
	14.13	8.80	5.33	37.72%
	17.12	17.67	−0.55	−3.21%
	19.20	18.94	0.26	1.35%
	8.34	6.10	2.24	26.86%
	9.45	10.2	−0.75	−7.94%
	2.31	2.05	0.26	11.26%
	3.56	3.82	−0.26	−7.30%
	21.32	20.21	1.11	5.21%
	23.79	22.33	1.46	6.14%
	5.09	6.21	−1.12	−22.0%
	8.07	7.21	0.86	10.6%
	10.25	12.21	−1.96	−19.1%
	17.94	18.21	−0.27	−1.51%
	21.74	23.21	−1.47	−6.77%
Transmission	11.11	11.56	−0.45	−4.05%
	14.13	14.78	−0.65	−4.60%
	17.12	17.13	−0.01	−0.06%
	19.2	19.11	0.09	0.47%
	8.34	8.47	−0.13	−1.56%
	9.45	9.21	0.24	2.54%
	2.31	2.21	0.10	4.33%
	3.56	3.51	0.05	1.40%
	21.32	20.91	0.41	1.92%
	23.79	23.82	−0.03	−0.13%
	5.09	5.24	−0.15	−2.94%
	8.07	8.49	−0.42	−5.20%
	10.25	10.15	0.1	0.98%
	17.94	19.55	−1.61	−8.97%
	21.74	19.21	2.53	11.60%

**Table 5:** Sample statistical results.

Sample	Number of Samples	Spectra	Qualitative		Quantitative	
			Calibration	Validation	Calibration	Validation
PPP	60	900	45	15	45	15
PPN	60	900	45	15		
PLP	60	900	45	15	45	15
PLN	60	900	45	15		

## Data Availability

The data presented in the study are available on request from the corresponding author. The data are not available due to privacy and ethical reasons.
